# Glucose-6-Phosphate Dehydrogenase Deficiency in an Endemic Area for Malaria in Manaus: A Cross-Sectional Survey in the Brazilian Amazon

**DOI:** 10.1371/journal.pone.0005259

**Published:** 2009-04-16

**Authors:** Marli Stela Santana, Marcus Vinícius Guimarães de Lacerda, Maria das Graças Vale Barbosa, Wilson Duarte Alecrim, Maria das Graças Costa Alecrim

**Affiliations:** 1 University of the State of Amazonas, Manaus, Amazonas, Brazil; 2 Tropical Medicine Foundation of Amazonas, Manaus, Amazonas, Brazil; 3 Nilton Lins Universitary Center, Manaus, Amazonas, Brazil; Instituto de Pesquisa Clinica Evandro Chagas, FIOCRUZ, Brazil

## Abstract

**Background:**

There is a paucity of information regarding glucose-6-phosphate dehydrogenase (G6PD) deficiency in endemic areas for malaria in Latin America.

**Methodology/Principal Findings:**

This study determined the prevalence of the G6PD deficiency in 200 male non-consanguineous individuals residing in the Ismail Aziz Community, on the outskirts of Manaus (Brazilian Amazon). Six individuals (3%) were deficient using the qualitative Brewer's test. Gel electrophoresis showed that five of these patients were G6PD A^−^. The deficiency was not associated with the ethnic origin (P = 0.571). In a multivariate logistic regression analysis, G6PD deficiency protected against three or more episodes of malaria (P = 0.049), independently of the age, and was associated with a history of jaundice (P = 0.020) and need of blood transfusion (P = 0.045) during previous treatment for malarial infection, independently of the age and the previous malarial exposure.

**Conclusions/Significance:**

The frequency of G6PD deficiency was similar to other studies performed in Brazil and the finding of a predominant G6PD A^−^ variant will help the clinical management of patients with drug-induced haemolysis. The history of jaundice and blood transfusion during previous malarial infection may trigger the screening of patients for G6PD deficiency. The apparent protection against multiple malarial infections in an area primarily endemic for *Plasmodium vivax* needs further investigation.

## Introduction

Malaria remains the most important tropical disease in tropical and sub-tropical areas of the world [1]. In Brazil, approximately 99.5% of malaria cases occur within the Amazon Region, with 544,615 in 2006. The State of Amazonas reported 181,973 cases, representing 33.4% of the total. In Manaus, the capital of the state, 51,228 cases were reported in that same year, and 40,679 (79.4%) of these were caused by *Plasmodium vivax* [2]. The continued high incidence of malaria in the municipality of Manaus is related to environmental and socio-economic factors that include high temperatures, humidity, vector density, unplanned expansion of the urban peripheral areas and resistance to antimalarials [3], [4].

To obtain the radical cure of vivax malaria, primaquine is prescribed for all patients [5]. Despite a low fatality rate, *P. vivax* infection may lead to severe clinical complications related to the side effects of the drugs, such as haemolysis induced by primaquine in individuals genetically deficient for G6PD. The worldwide distribution of this deficiency is a vital consideration in the development of new antimalarials that may also have the potential of causing haemolysis [6].

G6PD is an enzyme present in the cytoplasm of all cells, acting specifically in the maintenance of the integrity of the erythrocytes, preventing the oxidation of hemoglobin and other cellular proteins [7]. G6PD deficiency is X-linked and predisposes to hemolysis and to a lesser extent to methemoglobinemia in those persons in use of a substance with oxidative properties [8]. The degree of drug-induced hemolysis may be determined by the G6PD variants involved, grouped in five classes, identified according to the activity of the enzyme by electrophoretic and molecular characteristics [9], [10].

G6PD deficiency occurs most frequently in individuals of African descent, with a frequency ranging from 3.6 to 28.0% [11], [12]. In Asia the deficiency prevalence ranges from 6.0 to 15.8% [13], [14], in India it is 10.5% [15], and in the Middle East the prevalence varies from 3 to 29% [16], [17]. In Brazil, a few studies have found prevalences between 1.7 and 6.0% with a predominance of the African variant (A^−^), with new mutations already described only in non-endemic areas for malaria [18]–[21]. To date, there is no community-based estimate of the frequency of G6PD deficiency in highly-endemic areas for malaria in Latin America, such as Manaus, and the major variants predominating in this population are unknown. Therefore, this study aimed to estimate the prevalence of G6PD in an endemic area for malaria in Manaus, in the Brazilian Amazon.

## Results

Of the 200 analyzed samples, six (3%; CI95% 0.97–5.03%) were deficient for G6PD according to the first screening using the phenotypic Brewer's test. The deficiency was confirmed in all the samples by electrophoresis in agarose gel. Five samples with G6PD deficiency showed the type A^−^ standard on electrophoresis. One sample disclosed an indeterminate band in the gel, which did not permit a valid interpretation of the type of deficiency. All the patients were afebrile at the time of the thick blood smear collection, but three samples were positive for *Plasmodium* sp. (two for *P. vivax* and one for *P. falciparum*), disclosing an infection prevalence of 1.5% (CI95% 0.49–2.51). In Table 1, it is shown that G6PD was not associated with the ethnic origin, but was associated with less than three episodes of malaria throughout their lives and strongly associated with jaundice and need of blood transfusion during previous malarial infections. Table 2 shows that the same results were confirmed after a multivariate analysis (adjusting for age and previous malarial infections).

**Table 1:** Crude *odds ratios* and respective CI95% in the univariate logistic regression analysis of the association between G6PD deficiency and ethnic origin, previous malaria exposure and previous clinical complications triggered by malarial infection.

	Non-G6PD deficient n/N (%)	G6PD deficient n/N (%)	Total n/N (%)	OR (CI95%)	P
**Ethnic origin**					
White	25/194 (12.9)	1/6 (16.7)	26/200 (13.0)	-	-
Non-white	169/194 (87.1)	5/6 (83.3)	174/200 (87.0)	0.74 (0.08–17.43)	0.571*
**Previous malaria exposure**					
<3 episodes	86/194 (44.3)	5/6 (83.3)	91/200 (45.5)	6.27 (1.10–54.75)	0.041**
≥3 episodes	108/194 (55.7)	1/6 (16.7)	109/200 (54.5)	-	-
**Previous clinical complications triggered by malarial infection**					
Jaundice	48/163 (29.4)	5/6 (83.3)	53/169 (31.3)	11.98 (1.30–78.32)	0.012*
Need of blood transfusion	0/163 (0)	4/6 (66.7)	4/169 (2.3)	-	<0.001**

* χ^2^-test.

** Fisher exact test.

**Table 2:** Adjusted *odds ratios* and respective CI95% for the variables associated with G6PD deficiency in multiple logistic-regression models.

	OR (CI95%)	P*
**Previous malaria exposure** **		
<3 episodes	4.25 (1.05–60.82)	0.049
≥3 episodes	-	-
**Previous clinical complications triggered by malarial infection**		
Jaundice	7.70 (1.23–299.11)	0.020
Need of blood transfusion	-	0.045

* Modified Wilcoxon test for trend.

** Model 1: adjusted for age.

*** Model 2: adjusted for age and previous malarial exposure.

## Discussion

Since G6PD deficiency is a recessive trait linked to the X chromosome, studying the population frequency of the disorder exclusively in men is an optimal approach [6]. Measuring the deficiency in hospital-based studies may underestimate the true magnitude of the problem, because patients in a haemolytic crisis may increase their number of peripheral reticulocytes with normal G6PD values, thereby leading to a false-negative screening for G6PD deficiency.

The qualitative Brewer's test for the reduction of methemoglobin is a simple method that uses low-cost reagents. Although only yielding qualitative results, it is a good screening test and it has been widely utilized in various studies of selected populations [20], [22].

The biochemical characterization of the isoenzymes demonstrated a high frequency of the phenotype A^−^ G6PD deficiency. To a certain extent this confirms findings in other Brazilian studies with different study designs [22], [23]. Since one sample in our study could not be characterized through the biochemical approach, molecular studies are needed to confirm new possible variants in further studies. The common African variant G6PD A^−^ is usually a mild/moderate deficiency (10–15% of normal activity in hemizygous males). In this case, G6PD activity is greatest in younger cells, so as the red cell population recovers, G6PD activity increases and hemolysis is controlled. This is what makes drug-induced G6PD deficiency-related hemolytic anemia in G6PD A^−^ subjects a self-limiting problem in most cases [6]. Therefore, our study supports conservative management of hemolytic crisis for patients from Manaus, based on their predominant genotype.

Among the six individuals with the G6PD A^−^ variant, five were non-white (but not black) and one was white. This illustrates the impact of the racial mixing which occurred in the recent history of Brazil, especially in the Amazon region. A significant immigration from various parts of Brazil and abroad took place in the nineteenth century due to rubber extraction activities and during the twentieth century because of the creation of a tax-free zone and a growing industrial base in Manaus. The large number of immigrants from Portugal and Lebanon in Manaus probably explains this predominant variant found in the studied population, since G6PD A^−^ is frequently seen in these groups [7], [17], [18].

Epidemiologic studies have suggested that G6PD deficiency protects against malaria and severe malaria by *P. falciparum* [24]–[26]. The precise mechanism of protection remains unknown. We observed a significant protection against more than three episodes of malaria in the G6PD deficient men enrolled in this study, independently of their age, suggesting some degree of protection confered by the deficiency against *P. vivax* malaria, which is the major species in the area of the study. This potential protection conferred by G6PD deficiency needs further investigation.

The impact of G6PD deficiency in this population affected mostly by *P. vivax* infection is reflected in their history of significantly more jaundice and more blood transfusions. This information could be useful as a first screening for G6PD deficient patients, to determine who should have a Brewer's test performed. This test is not routinely offered to the general population with a diagnosis of *P. vivax* malaria in Brazil even though they will receive a standard 7-day treatment regimen with primaquine (0.5 mg/kg/day) for radical cure of the parasite. G6PD deficiency therefore increases the morbidity of *P. vivax* infection itself and the side effects of primaquine. In both cases the costs of treatment and hospitalization are increased. These additional expenses threaten financial resources and difficulty achieving a radical cure complicates the control of *P. vivax* malaria.

Since only a few studies have been published estimating the prevalence of G6PD deficiency in malaria endemic areas of Latin America, this study adds information to the global mapping of the problem, despite a low external validity, as most of the literature on this issue. Understanding the burden of G6PD deficiency will guide local antimalarial treatment policies and enable the proper design of clinical trials for *P. vivax* radical cure with new antimalarials having the potential of hemolysis in this population, such as tafenoquine [27].

Despite the small number of patients enrolled in this population-based survey, we detected a 3% prevalence of G6PD deficiency in an endemic area for malaria in Manaus. Accurate information about this deficiency and its relation to acute hemolysis with the use of primaquine in the Brazilian Amazon is scarce. G6PD A^−^ was confirmed through electrophoretic isoenzyme detection in 5 of 6 patients after the deficiency was initially detected via screening with the qualitative Brewer's test. G6PD A^−^ was not associated with a black ethnicity, probably because of the genetically mixed population of Manaus. The history of jaundice and blood transfusions associated with prior malaria infections was associated with G6PD deficiency and may be used as a simple clinical marker for this entity.

## Materials and Methods

**Figure 1:**
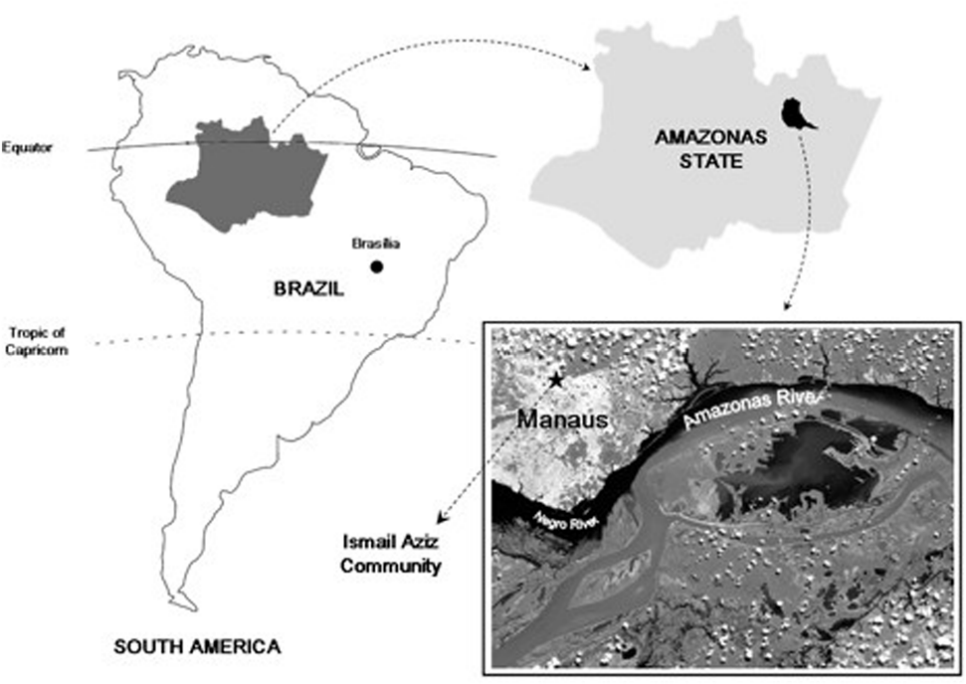
Geographic localization of the Ismail Aziz Community in an endemic area for malaria in Manaus, Amazonas State, Brazil.

The study was carried out in the municipality of Manaus (03°08′ S/60°00′ W), in the Ismail Aziz Community, with an estimated population of 1,500 inhabitants (750 male), including natives and immigrants (Figure 1). Census data were used to estimate the population size. The community was chosen due to a high annual parasite index in 2006 (234/1,000 persons) [2]. The sample size was calculated for a population of 750 non-consaguineous men (in order to avoid the consanguinity bias) with an expected prevalence of G6PD deficiency of 3%, power of 80% and significance level below 5%. Therefore, 200 non-consanguineous men between 1 and 65 years-old (children under one year are physiologically G6PD deficient and over sixty-five, due to the decrease of enzyme activity during senile) were randomly selected (selection of the fourth man in the visit to all the houses of the community, except those who were consaguineous relatives of other selected subjects) and enrolled in a cross-sectional survey of G6PD deficiency, after informed consent has been obtained. Ethnic origin, previous malaria infection and previous clinical complications triggered by malarial infection were acquired through an individual interview, and all the data were recorded in a standard questionnaire.

### Laboratorial techniques

Ten milliliters of venous blood were collected from each person using EDTA tubes (BD Vacutainer®). A qualitative G6PD screening test by the methemoglobin reduction method was performed [28]. In all those initially found to be G6PD deficient, after the whole blood samples were hemolysed, the material was submitted to electrophoresis [29] for the confirmation of the deficiency by isoenzyme analysis. The diagnosis of the malaria was based in the method of the thick blood smear by Walker and examinated directly to the optic microscope, according to norms of the World Health Organization, was carried through in all the individuals [30].

### Statistics

The statistical analyses were performed in Epi Info® 3.3 2004 (CDC/Atlanta). 95% CIs (CI95%) were estimated for all the observed frequencies of the study. To assess the extent to which ethnic origin, previous malaria exposure and previous clinical complications triggered by malarial infection were associated with G6PD deficiency, *odds ratios* (OR) with 95% CIs (CI95%) were estimated by univariate logistic regression analysis (Table 1, Table 2). Qualitative data were analyzed with the χ^2^-test or the Fisher exact-test when indicated. Adjustment for multiple variables was performed by adding the covariates in a set of multiple logistic-regression models. Test for trend was conducted through the exact test for trend (modified Wilcoxon test for trend). A two-tailed value of P<0.05 was considered statistically significant.

### Ethical clearance

The study was approved by the Ethics Committee Board of the Tropical Medicine Foundation of Amazonas. Informed written consent was obtained from all participants.
